# New Insights Into DNA Helicases as Druggable Targets for Cancer Therapy

**DOI:** 10.3389/fmolb.2018.00059

**Published:** 2018-06-26

**Authors:** Arindam Datta, Robert M. Brosh

**Affiliations:** Laboratory of Molecular Gerontology, National Institute on Aging, National Institutes of Health, NIH Biomedical Research Center, Baltimore, MD, United States

**Keywords:** helicase, DNA repair, replication, genomic instability, small molecule, therapy, cancer

## Abstract

Small molecules that deter the functions of DNA damage response machinery are postulated to be useful for enhancing the DNA damaging effects of chemotherapy or ionizing radiation treatments to combat cancer by impairing the proliferative capacity of rapidly dividing cells that accumulate replicative lesions. Chemically induced or genetic synthetic lethality is a promising area in personalized medicine, but it remains to be optimized. A new target in cancer therapy is DNA unwinding enzymes known as helicases. Helicases play critical roles in all aspects of nucleic acid metabolism. We and others have investigated small molecule targeted inhibition of helicase function by compound screens using biochemical and cell-based approaches. Small molecule-induced trapping of DNA helicases may represent a generalized mechanism exemplified by certain topoisomerase and PARP inhibitors that exert poisonous consequences, especially in rapidly dividing cancer cells. Taking the lead from the broader field of DNA repair inhibitors and new information gleaned from structural and biochemical studies of DNA helicases, we predict that an emerging strategy to identify useful helicase-interacting compounds will be structure-based molecular docking interfaced with a computational approach. Potency, specificity, drug resistance, and bioavailability of helicase inhibitor drugs and targeting such compounds to subcellular compartments where the respective helicases operate must be addressed. Beyond cancer therapy, continued and new developments in this area may lead to the discovery of helicase-interacting compounds that chemically rescue clinically relevant helicase missense mutant proteins or activate the catalytic function of wild-type DNA helicases, which may have novel therapeutic application.

## Introduction

Targeting the DNA damage response and DNA repair to combat cancer became an attractive hypothesis with the original discoveries made by Thomas Helleday, Alan Ashworth and colleagues that chemicals which inhibit the DNA damage sensor poly(ADP-ribose) (PAR) polymerase 1 (PARP-1) could be used to kill breast cancer cells that are defective in the tumor suppressor genes encoding homologous recombination (HR) repair proteins BRCA1 or BRCA2 (Bryant et al., 2005; Farmer et al., 2005). As elaborated below, there has been much interest in the mechanisms of PARP inhibitors as well as topoisomerase inhibitors used in preclinical and clinical settings, and the progress made in these areas have prompted biomedical researchers to investigate these and other potential therapeutic DNA repair proteins as targets to enhance the effects of chemotherapy drugs or ionizing radiation to eradicate cancer cells but spare normal cells, thereby minimizing toxicity usually associated with the DNA damaging treatments. An important aspect of small molecule drugging of at least some DNA repair protein targets involves trapping the enzyme on its DNA substrate resulting in a poisonous protein complex, which will be discussed as a possible precedent for a new class of chemical inhibitors that target DNA helicases, the subject of this review.

DNA helicases are ubiquitous enzymes found in all domains of life and involved in every aspect of nucleic acid metabolism (Crouch and Brosh, 2017). As molecular motors, they utilize the energy derived from binding and hydrolysis of nucleoside triphosphate (typically ATP) to translocate on DNA and disrupt the many hydrogen bonds between complementary strands of the DNA double helix. In addition, certain DNA helicases unwind alternate DNA structures such as triplexes or G-quadruplexes and/or displace proteins bound to single-stranded or double-stranded DNA. DNA helicases play instrumental roles in cellular DNA replication, transcription, DNA repair, and other processes to preserve genomic integrity and maintain cellular homeostasis. Their vital functions are illustrated by the fact that mutations in a number of helicase genes are either linked to hereditary diseases characterized by chromosomal instability or associated with various cancers (Brosh, 2013).

The molecular differences among helicases are of interest as they may provide opportunities for targeting specific helicases in anti-cancer therapies. DNA helicases are broadly categorized according to the grouping (Superfamily (SF)/Family) to which they belong based on sequence homology within conserved motifs in the helicase core domain as well as auxiliary domains residing in the N- or C-terminal regions of the protein (Umate et al., 2011). Many of the human DNA repair helicases belong to SF2, and the two most prominent families are the RecQ helicases and Iron-Sulfur (Fe-S) helicases which have opposite polarities of single-stranded DNA translocation (Estep and Brosh, 2017). The 3′ to 5′ RecQ helicases (RECQL1, WRN, BLM, RECQL4, RECQL5) share prominent roles in replication fork remodeling, double-strand break (DSB) repair, and regulation of gene expression mediated by their nucleic acid structure-specific binding and catalytic properties as well as their protein interactions. Molecular defects in the RecQ helicases give rise to genomic instability, and mutations in WRN, BLM, and RECQL4 are linked to hereditary diseases characterized by accelerated aging or associated with cancer (Croteau et al., 2014). All five human RecQ helicases are up-regulated in various cancers, suggesting their specialized requirement in rapidly dividing cells to repair replicative lesions or elicit an appropriate response in cell cycle checkpoint or gene expression (Brosh, 2013). As discussed further in the review, structural characterization of various RecQ helicases has provided new insight to the functional importance of key structural elements within the helicase core as well as auxiliary regions that may lead to the design of small molecules which target specific domains.

Given their crucial roles in DNA replication, repair, and genomic stability, DNA metabolic proteins with a characteristic Fe-S cluster have attracted interest from both the basic science and clinical perspectives. Apart from several DNA repair proteins (e.g., DNA glycosylases) and DNA polymerases, certain DNA helicases and helicase-nuclease enzymes possess a conserved Fe-S cluster domain (Wu and Brosh, 2012). The presence of a Fe-S cluster in DNA helicase enzymes was first discovered in XPD, the founding member of a group of DNA repair helicases (DDX11, RTEL-1, FANCJ) that unwind duplex DNA with 5′-3′ polarity and are implicated in human chromosomal instability disorders (Rudolf et al., 2006). Research from several labs established that the Fe-S cluster is essential for DNA unwinding by XPD and other Fe-S helicases (Estep and Brosh, 2017), including FANCJ (Wu et al., 2010).

In addition to the SF2 helicases, the SF1 Pif1 helicase is thought to play an important role in nuclear DNA replication (Budd et al., 2006), telomere replication/repair (Geronimo and Zakian, 2016), and mitochondrial DNA synthesis (Lahaye et al., 1991; Pinter et al., 2008). Pif1 may serve to aid the 5′ to 3′ Twinkle hexameric ring-like helicase (SF4) as it generates single-stranded DNA template through difficult-to replicate sequences (Korhonen et al., 2003). For nuclear DNA replication, the ring-like 3′ to 5′ helicase complex constituted by the MCM2-7 proteins (SF6) is essential (Chong et al., 2000). The ring-like structures of the replicative helicases Twinkle (Fernandez-Millan et al., 2015) and MCM complex (Zhai and Tye, 2017), combined with their accessory factors [e.g., mitochondrial single-stranded binding protein (Korhonen et al., 2003), Cdc45/GINS (Aparicio et al., 2009)], enhance the processivity of these helicases to fulfill unwinding of duplexes inherently longer than what is required for strand separation by the helicases implicated in stalled fork remodeling or DNA repair.

**Figure 1:**
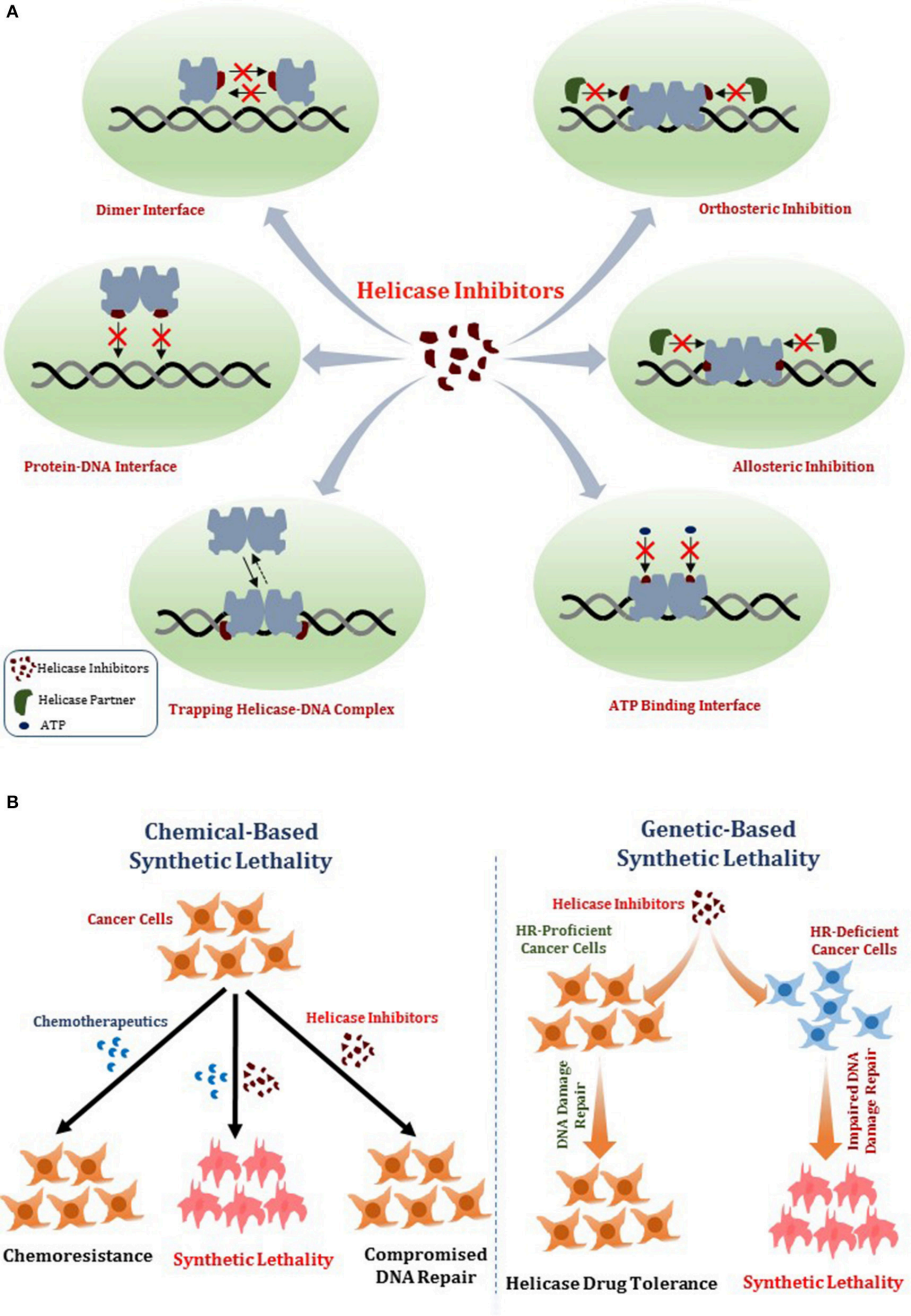
Mechanisms of DNA helicase inhibitors and therapeutic strategies**. (A)** Small molecule helicase inhibitors may interfere with the catalytic activities of DNA helicase proteins and their molecular and cellular functions by a variety of mechanisms. A helicase-interacting compound may disrupt protein oligomerization, binding to DNA substrate, or compete with ATP binding. Small molecules may alter helicase interactions with other proteins (e.g., DNA repair/replication factors) by orthosteric or allosteric mechanisms. Helicase-interacting compounds may also cause the protein to become trapped on DNA, resulting in a toxic complex or lead to the hijacking of other proteins. **(B)** Two potential strategies for helicase inhibitors (that are not mutually exclusive) are (i) Chemical-based synthetic lethality whereby pharmacological helicase inhibition compromises the cancer cell to chemotherapy DNA damaging drugs or radiation; (ii) Genetic-based synthetic lethality whereby the defined genetic mutant background of the cancer cell is hypersensitive to pharmacological helicase inhibition. See text for details.

**Figure 2:**
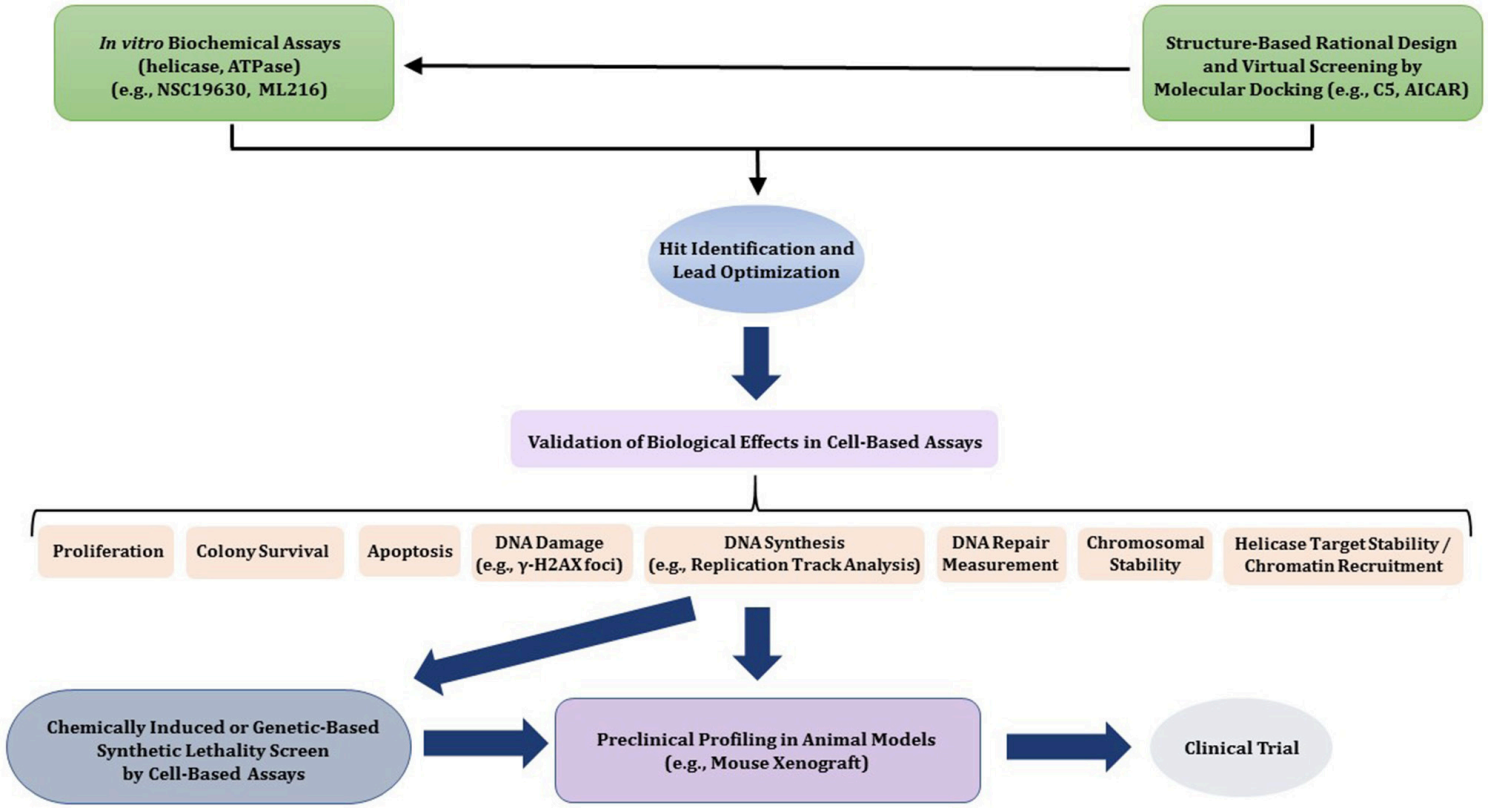
Flow diagram for discovery, optimization, and validation of DNA helicase inhibitors. See text for details.

In this review, we will provide a framework for thoughtfully considering DNA helicases as a desirable new avenue to target for anti-cancer therapy. In Figure 1, we depict some potential modes of small molecule inhibition of DNA helicases, as well as chemically induced or genetic synthetic lethality that will be referred to in the text. Clearly, an intricate molecular knowledge of helicase conformational states, substrate specificities, protein interactions, pathways, etc. is required to screen for compounds which target helicases successfully *in vitro* and *in vivo* with optimal characteristics. We will discuss novel and emerging concepts and developments in anti-cancer therapy as they relate to proposed helicase targets, highly relevant to the prognosis of individuals suffering from many types of cancer that remain a major health risk and source of mortality. Moreover, the current anti-cancer strategies are still highly sub-optimal in many treatments due to the toxicity in normal cells and tissues imposed by chemotherapy drugs and radiation. With the advent of new helicase inhibitors discovered by both high-throughput *in vitro* assays and *in silico* compound screening approaches relying on molecular docking, the stage is set to assess their efficacy using preclinical *in vivo* models (Figure 2).

## DNA damage response proteins: targets for cancer therapy?

The concept of DNA repair or replication stress response modulation for therapeutic intervention has become a hot topic of research and in recent years, clinical pursuit. The field really got its start with the discovery of PARP inhibitors and topoisomerase inhibitors and has taken off with the identification and characterization of novel DNA repair targets. This discussion provides an excellent backdrop for consideration of DNA helicases as potential targets for chemical modulation. From a clinical perspective, personalized medicine has become prominent over the past decade or more. Understanding the genotype-phenotype relationships controlling tumor aggressiveness and their influence over the effectiveness of chemotherapy/radiation treatments has become of increasing importance to the emerging field of DNA damage signaling and DNA repair inhibitors (Velic et al., 2015; Hengel et al., 2017). As illustrated above by the discussion of PARP and topoisomerase inhibitors, their efficacy to combat cancer is dependent on the genetic background of the tumor.

### Seminal discovery of PARP inhibitors

Over a decade ago, the concept of DNA repair inhibition emerged in the laboratory setting as a potential avenue for the development of DNA damage response or DNA repair inhibitors with the discovery of small molecules (< 300 Da) that deter the molecular and cellular function of PARP (Bryant et al., 2005; Farmer et al., 2005). PARP inhibitors impair the enzyme's ADP-ribose modification function, which in turn suppresses its role in base excision repair, single-strand break repair, and more generally, DNA damage sensing (Cseh et al., 2017). In the case of PARP-1 inhibitors, studies were historically focused on familial and sporadic breast and ovarian cancers with bi-allelic mutations in the HR repair genes *BRCA1, BRCA2*, or *PALB2* (Hengel et al., 2016; Pommier et al., 2016). The rationale was that pharmacological inhibition of PARP-1 function in rapidly dividing cancer cells would result in an accumulation of single-strand breaks leading to broken replication forks that are essentially DSBs. These DSBs that normally would be repaired by HR with the sister chromatid duplex would be fixed inefficiently in certain cancers due to the absence of any one of the three key HR repair proteins BRCA1, BRCA2, or PALB2. Although some success was achieved for treatment of ovarian cancer patients with the PARP-1 inhibitor olaparib, resistance to the drug has limited its therapeutic effectiveness (Murata et al., 2016), leaving researchers to continue exploring and developing new and better cancer therapies focused on PARP inhibitors targeting each member of the family and optimal co-treatment strategies of these compounds with other anti-cancer agents.

### Development of clinically relevant topoisomerase inhibitors

Topoisomerase inhibitors are perhaps as high profile as the PARP inhibitors for potential clinical use (Pommier et al., 2010). The discovery and development of topoisomerase inhibitors that cause cytotoxicity in cancer cells has sparked tremendous interest in their suitability for anti-bacterial and anti-cancer applications. Much has been learned about the mechanism of action of topoisomerase-poisoning inhibitors, and it is postulated that many of these compounds act in a similar manner to certain PARP inhibitors by trapping the enzyme on DNA (see below).

Researchers are engaged in the quest to discover more effective topoisomerase (as well as PARP) inhibitors that can hit every cellular target, are chemically stable and behave optimally according to pharmacokinetic parameters. In addition, chemotherapy drug combinations need to be optimized. Aside from these challenges, the looming concerns for compounds that impair the functions of other DNA repair proteins is their effective targeted drug delivery and sub-optimal therapeutic index (Hengel et al., 2017). Further studies that elucidate the pathways whereby such inhibitors act in cells to exert their cytotoxicity and optimize tumor-specific delivery approaches are high priorities in the field. Moreover, combination therapies that exploit the genomic signature of a tumor may lead to the development of anti-cancer strategies which lower the cancer-killing drug doses, thereby sparing normal cells and tissues. Such efforts in precision medicine have become paramount (O'Connor, 2015).

### Trapping PARP- and topoisomerase-DNA complexes presents a paradigm for new anticancer drugs

Foreshadowing the potential mechanism of action of DNA helicase inhibitors (discussed below), research has revealed that some chemical PARP inhibitors and topoisomerase inhibitors act by trapping the enzyme on DNA, thereby poisoning cells via the formation of toxic DNA-protein-drug complexes that have consequences beyond simply inhibiting catalytic function (Pommier et al., 2015). Yves Pommier and colleagues first used the term interfacial inhibitors to describe drugs that trap protein-DNA complexes by binding at their interfaces, and the concept has been expanded to include medicinal compounds that bind at protein-protein interfaces as well (Pommier and Cherfils, 2005). An excellent example of the former, highly germane to certain emerging DNA repair inhibitors, is represented by those compounds which inhibit topoisomerase action by binding to the very site where the enzyme interacts with DNA to cleave its phosphate backbone, resulting in a trapped topoisomerase inhibitor-stabilized cleavage complex (Pommier, 2013).

In parallel to such interfacial topoisomerase inhibitors, certain PARP-1 and PARP-2 inhibitors act by trapping PARP on DNA *in vitro*, helping to explain why cellular exposure to these PARP-binding drugs exerts a greater cytotoxicity than the absence of PARP altogether (Pommier et al., 2016). The development of PARP trapping assays using extracts of PARP inhibitor-treated cells to assess chromatin enrichment or employing purified PARP recombinant proteins and fluorescently or radioactively labeled oligonucleotide-based DNA substrates with site-specific damage (e.g., single-strand nick) incubated with PARP inhibitor has provided seminal evidence for DNA-bound PARP complexes. From a more clinical perspective, there is great interest in understanding the mechanisms for resistance to PARP inhibitors such as PARP expression, drug efflux, and changes in DNA damage response/DNA repair in response to PARP inhibitor exposure. In addition, ongoing efforts in anti-cancer therapy focus on developing successful combinations of PARP inhibitor with other DNA damaging treatments and/or DNA repair inhibitors. Studies of the interfacial PARP inhibitors and topoisomerase inhibitors will likely serve as models for the future investigations of DNA helicase inhibitors that behave according to a similar enzyme trapping mechanism; however, these mechanistic analyses are only in their infancy (see below).

### New targets in genomic DNA metabolism to enhance cancer therapy

While topoisomerase and PARP inhibitors continue to attract interest as targets for anti-cancer therapy, in recent years other DNA damage response and DNA repair targets have emerged. For the small molecules that inhibit these proteins, some of the same basic idealized principles apply in which the chemical agents acting as a monotherapy or in combination with other chemotherapy treatments will target the cancerous cells and tissues, sparing normal ones by exploiting a therapeutic threshold index. Some examples of new DNA repair inhibitors that target HR repair proteins (e.g., RAD51, RAD52, RAD54), the structure-specific nuclease MRE11, and others are summarized in recent reviews (Huang and Mazin, 2014; Velic et al., 2015; Hengel et al., 2017). These relatively new targets, like the PARP- and topoisomerase-interacting drugs, may provide insights to mechanistic aspects of helicase inhibitors and their application in anti-cancer regimes. For example, small molecule inhibitor-induced trapping of DNA metabolic proteins may represent a more generalized mechanism with poisonous consequences applicable to compounds that target other DNA repair proteins such as DNA methyltransferases and DNA helicases.

## DNA repair inhibitors identified by high-throughput screens and molecular docking approaches

As mentioned above, small molecule inhibitors of proteins implicated in DNA damage signaling and DNA repair identified by high-throughput screens (HTS) have been advanced by basic research efforts in part to develop anti-cancer strategies to enhance chemotherapy or radiation treatments. These assays can be broadly divided into two categories: (1) Biochemical screens which are used to directly assess modulation of protein function, be it enzymatic activity or ligand binding; (2) Cell-based assays used to investigate if a set of compounds influence a DNA repair pathway, ultimately with an outcome on cellular homeostasis and/or genomic stability. Often, once a compound is identified that is potent and specific for its target *in vitro*, chemists will optimize its structural and solubility properties for *in vivo* application.

In the following sections, we will highlight some examples of DNA repair inhibitors and DNA helicase inhibitors discovered by HTS and molecular docking approaches. Practically all the presently known helicase inhibitors have been shown to act synergistically in a genetic or chemical manner with druggable DNA repair targets in cell-based systems, and some of these synergistic combinations will be discussed.

### Structure-based design of DNA repair inhibitors by molecular docking approaches

Although significant advancement has been made in developing potential small molecule inhibitors targeting DNA repair machinery, only a few have reached the clinic so far. Hits primarily identified by HTS based on *in vitro* biochemical assays sometimes fail to exert their desired effect at the cellular level and are often non-specific. In general, the associated cost, time, assay complexities and screening quality are considered as major challenges in developing highly potent and specific drug-like molecules using experimental HTS approaches (Shoichet et al., 2002; Moitessier et al., 2008; Awate and Brosh, 2017). With the rapid advancement in computational methodologies coupled with the availability of high-resolution crystal structures of target proteins, structure-based virtual screening of large compound libraries has drawn significant attention in modern drug discovery research over the last two decades. The approach has been successfully used to identify highly accurate lead molecules in a time- and cost-effective manner (Kroemer, 2007; Meng et al., 2011). Among the various structure-based *in silico* compound screening methodologies, molecular docking technique is widely adopted and considered as the principal one. Given the three-dimensional structure of the target protein, this important computational tool allows the researchers to virtually screen a large set of small organic molecules and provides information about the binding mode and strength of the binding for individual protein-ligand complexes. Therefore, molecular docking is useful not only in identifying new hits but also in facilitating the further optimization of the pre-identified lead molecules to develop more potent analogs.

## Small molecule inhibitors of DNA helicases

Although somewhat lagging behind in the field of small molecule DNA repair inhibitors, pharmacological inhibition of DNA helicases has begun to attract interest. A recent review summarizes experimental approaches to identify and characterize DNA helicase inhibitors by biochemical and cell-based assays (Banerjee et al., 2016). With the discovery of new helicase protein structures and a growing understanding of their molecular mechanisms, there has been increasing interest in small molecules that modulate helicase function. Below, we provide the reader a current assessment of the field. Given the number of both DNA and RNA helicases implicated in fundamentally important areas of nucleic acid metabolism in human cells, it seems likely that continued advances in pharmacological interventions will be made. These advances should provide unique tools to investigate the cellular functions of helicases, their biological pathways in nucleic acid transactions, and further development in pre-clinical models (Figure 2).

While some helicase inhibitor studies have focused on pharmacological modulation of human DNA helicases involved in DNA damage responses that would affect the efficacy of ionizing radiation or chemotherapy treatments, a number of inhibitors of viral helicases have been discovered over the past decade that may be useful for suppressing viral diseases (Shadrick et al., 2013). The clinical success of a herpes simplex virus (HSV) helicase-primase complex inhibitor known as Amenamevir (ASP2151) is a strong testament to viral helicases as potential druggable targets to deter viral pathogenesis (Chono et al., 2010; Katsumata et al., 2012; Tyring et al., 2012). ASP2151 has a broad anti-herpes virus spectrum including HSV-1, HSV-2, and varicella zoster virus as well as acyclovir-resistant thymidine kinase–deficient HSV strains (Chono et al., 2010; Himaki et al., 2012). Although not yet clinically proven, small molecule-based approaches showed significant progress in targeting other viral helicases such as human papilloma virus (HPV) and hepatitis C virus (HCV) helicases. By high-throughput screening and subsequent chemical optimization, a family of biphenylsulfonacetic acid-based small molecules was discovered that inhibit the ATPase and helicase activity of HPV6 E1 helicase *in vitro*, but their bioactivity remains elusive (Faucher et al., 2004). On the other hand, a novel class of small molecules that specifically antagonizes the physical interaction of HPV E1 helicase with E2 protein was found to be effective to inhibit HPV DNA replication in cell-based assays (White et al., 2003; Yoakim et al., 2003). This work serves as an excellent example of targeting viral helicase protein-protein interactions to develop potential antiviral therapies. As reviewed elsewhere, the hepatitis C virus NS3 helicase is considered a potential candidate for specifically targeted antiviral therapy (Belon and Frick, 2009). Lead compounds (e.g., Soluble blue HT, Acridone-4-carboxylic acid derivatives, benzotriazoles) that inhibit HCV helicase activity and impair cellular HCV RNA replication hold immense potential. For a comprehensive review of new and developing antiviral and antibiotic small molecule inhibitors, see (Shadrick et al., 2013).

### *In vitro* and cell-based properties of known compounds that inhibit DNA unwinding catalyzed by human helicases

Table 1 lists recently identified small molecular inhibitors of human DNA helicases. Most of the small molecule helicase inhibitors were identified from *in vitro* helicase assays using purified recombinant DNA helicase proteins, oligonucleotide-based DNA substrates, and small molecule libraries. However, a virtual screen of FDA-approved drugs by a nuclease-based assay identified an inhibitory compound for the DNA2 helicase-nuclease implicated in Okazaki fragment processing during DNA replication (Liu et al., 2016). Of those tested in cell-based assays, small molecule inhibitors of DNA unwinding catalyzed by WRN (Aggarwal et al., 2011, 2013b), BLM (Nguyen et al., 2013), and DNA2 (Liu et al., 2016; Kumar et al., 2017) all negatively affect proliferation of cancer cells and induce DNA damage and/or chromosomal instability. Moreover, these helicase inhibitors behave synergistically with other compounds that induce DNA damage, inhibit DNA repair, or impose replication stress. Certain helicase inhibitors operate in a manner that is dependent on the presence of the DNA helicase target (Aggarwal et al., 2011, 2013b; Nguyen et al., 2013; Liu et al., 2016), suggesting that pharmacological inactivation of helicase function involves the interference of a genome maintenance pathway which is distinct from the effect imposed by the absence of the helicase altogether. Presumably, backup mechanisms are elicited in certain helicase-deficient backgrounds, whereas a helicase inhibitor complex with its target imposes uniquely deleterious effects, akin to those caused by a protein trapping mechanism discussed earlier. While an inhibitor of the RECQL1 helicase was identified from an *in vitro* helicase activity screen (https://pubchem.ncbi.nlm.nih.gov/bioassay/2708), no published advances in terms of effects of the small molecule on functionality or metabolism of human cells have been reported. In the following sections, we will discuss some key features of the human DNA helicase inhibitor studies to provide the reader a sense of the field's current advances and future directions with an eye on clinical applications.

**Table 1:** Small molecule inhibitors of human DNA helicases.

**Helicase/compound**	**Screen/library**	***In vitro*** **properties**		**Biological effects**	**References**
		**Biochemical effects**	**Specificity^a^**		
WRN NSC19630 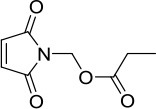	Helicase screen; NCI Diversity Set	IC_50_ ~ 20 μM; Inhibits DNA unwinding	Helicase-specific	Bioactive at 1–3 μM; Inhibits proliferation; Induces DNA damage; Synergistic with PARP inhibitor, CPT, or TMS; WRN-dependent	Aggarwal et al., 2011
WRN NSC617145 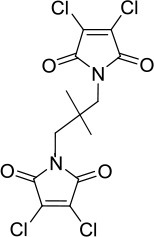	Helicase screen; NCI Diversity Set	IC_50_ ~230 μM; Inhibits DNA unwinding	Helicase-specific	Bioactive at 0.125–1 μM; Inhibits proliferation; Induces DNA damage; Synergistic with MMC in FA mutant background; WRN-dependent	Aggarwal et al., 2013b
BLM ML216 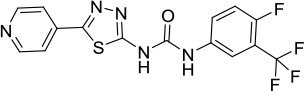	Helicase screen; MLSMR	IC_50_ ~ 3 μM; Impairs DNA binding, helicase	Inhibits WRN helicase	Bioactive at 50 μM; Inhibits proliferation; Elevates SCE; Synergistic with aphidicolin; BLM-dependent	Nguyen et al., 2013
DNA2 C5^b^ 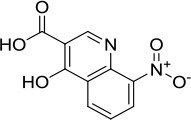	Virtual screen of FDA drugs; Nuclease screen; NCI DTP Set	IC_50_ ~ 20 μM; Impairs DNA binding, helicase, nuclease	Nuclease-specific; DNA2 helicase not assessed	Bioactive at 7–70 μM; Inhibits proliferation, fork resection, and recombination; Alters fork restart in BRCA2 / BOD1L mutant background; Synergistic with PARP inhibitor; DNA2-dependent	Liu et al., 2016
DNA2 NSC-105808 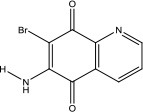	Nuclease screen; NCI DTP Set	IC_50_ ~ 2 μM (yeast DNA2); IC_50_ ~ 1.49 μM (human DNA2); Inhibits nuclease activity	Nuclease-specific; Helicase not assessed	Bioactive at 0.25–2 μM; Inhibits HR repair, DSB end resection and suppresses proliferation of cancer cells	Kumar et al., 2017
DDX3 Rhodamine-based derivative 4 (RBD4) 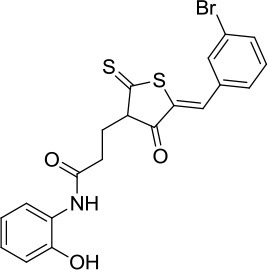	Virtual screen	Inhibits DDX3 ATPase activity (IC_50_ = 5.4 μM)	Helicase not assessed	Inhibits HIV-I (III_B_) replication in MT-4 leukemia cells (EC_50_ = 86.7 μM)	Maga et al., 2008
DDX3 Compound 1 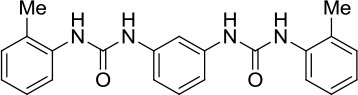 Compound 6 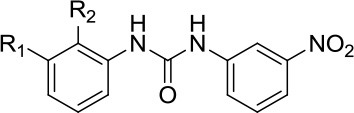 Compound 8 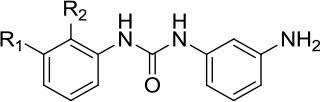	Virtual screen	Inhibits DDX3 ATPase (compound 1, IC_50_ = 17 ± 2 μM; compound 6, IC_50_ = 20 ± 3 μM; compound 8, IC_50_ = 40 ± 0.5 μM) and helicase activities (Compound 1, IC_50_ = 65 ± 5 μM; compound 6, IC_50_ = 1 ± 0.2 μM; compound 8, IC_50_ = 5 ± 0.6 μM)	Helicase not assessed	Suppresses HIV-1 replication in ^b^PBMCs (compound 6, EC_50_ = 10 μM; compound 8, EC_50_ = 15 μM)	Radi et al., 2012
Mcm4/6/7 Heliquinomycin 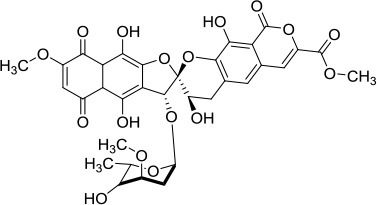	Helicase screen	IC_50_ ~ 2.4 μM; Inhibits DNA unwinding	Helicase-specific	Bioactive at 2–14 μM; Inhibits proliferation of cultured cancer cells	Ishimi et al., 2009; Toyokawa et al., 2011
Mcm2-7 Ciprofloxacin 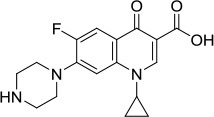	Helicase screen	IC_50_ ~ 632 μM; Inhibits DNA unwinding	Helicase-specific	Bioactive at 520–670 μM (yeast) and 160–350 μM (human cells); Inhibits proliferation of yeast and human cells	Simon et al., 2013

a *Specificity determined by its effect on other DNA helicases*.

b *DNA2 nuclease, but not helicase activity, was assessed*.

### WRN

Bi-allelic mutations in the *WRN* gene result in Werner syndrome (WS), a progeroid disease considered by many to most closely resemble accelerated aging (Oshima et al., 2017). With the apparent exception of neurodegeneration, almost all clinical features associated with normal aging (e.g., heart disease, osteoporosis, diabetes, cataracts, etc.) are observed early in life (concomitant with the adolescent growth spurt) for those individuals diagnosed with WS. Although the *WRN* gene product has been studied by molecular biologists, biochemists, cell biologists, and clinicians for over 2 decades, it is still unclear the molecular defects of WRN that are responsible for the mutant cellular phenotypes which include chromosomal instability, replication and DNA damage response defects, and abnormal transcriptional regulation. The *WRN* gene encodes a protein that has both DNA helicase activity and DNA exonuclease activity. Moreover, WRN interacts with a large cast of proteins implicated in various DNA transactions important for DNA repair, the replicational stress response, and telomere capping, suggesting that it may have pleiotropic roles. Although WRN mutant mouse models, particularly those crossed with other mutant mice, have provided some clues to WRN's involvement in telomere metabolism (Chang et al., 2004; Du et al., 2004; Laud et al., 2005), the definitive molecular and cellular deficiencies underlying WS remain elusive.

To provide a fresh approach to studying WS, we conducted a search for small molecules that behaved as potent and specific WRN helicase inhibitors (Aggarwal et al., 2011). The *in vitro*-based compound screen using a conventional biochemical DNA unwinding assay was performed to identify inhibitors of purified recombinant human WRN helicase-catalyzed DNA unwinding and positive hits were tested in cell-based assays. These efforts led to the identification and characterization of the compound NSC 19630 from the National Cancer Institute (NCI) Diversity Set which inhibited WRN-catalyzed DNA unwinding of a forked duplex DNA substrate (IC_50_ ~ 20 μM) in a specific manner based on the observation that other purified recombinant DNA helicase proteins tested (e.g., BLM, RECQL1) were either not inhibited or only modestly affected at much greater compound concentrations (Table 1). The inhibitory effect of NSC 19630 on WRN DNA unwinding was not mirrored by a similar effect on DNA binding or ATP hydrolysis, suggesting that the compound specifically interfered with WRN's strand separation activity. Biological studies with NSC 19630 and the human cervical cell line HeLa demonstrated that the WRN helicase inhibitor negatively affected cell proliferation and replication, as well as induced DNA damage and apoptosis in a WRN-dependent manner based on the observation that the same cells depleted of WRN by RNA interference were resistant to the effects of NSC 19630. Further studies showed that NSC 19630 behaved synergistically with the topoisomerase inhibitor topotecan, the PARP1 inhibitor KU0058948, or the G-quadruplex binding ligand Telomestatin for inhibiting proliferation and inducing DNA damage (Aggarwal et al., 2011), suggesting that pharmacological inhibition of WRN helicase activity under conditions of replicational stress or DNA damage severely compromised the cellular response.

In subsequent work, a compound designated NSC 617145 that is structurally related to NSC 19630 was found to inhibit WRN helicase activity with even greater potency (IC_50_ ~ 230 nM) and render human cells deficient in the Fanconi Anemia (FA) pathway hypersensitive to the DNA cross-linking agent mitomycin C (MMC) in a WRN-dependent manner (Aggarwal et al., 2013b) (Table 1). Interestingly, NSC 617145 did not sensitize FA-deficient cells to hydroxyurea (HU) (which causes replication stress by depleting the deoxynucleotide phosphate pool), suggesting that DNA DSBs that accumulate when the FA pathway is crippled in its ability to respond to interstrand cross-links (ICL) are particularly problematic in the face of poor WRN helicase activity as opposed to simply stalled forks. Perhaps WRN may aid in the repair of such DSBs by mediating HR. Supporting the hypothesis that the interaction of the helicase inhibitor with WRN causes the formation of a toxic ternary complex with genomic DNA that interferes with normal DNA repair, NSC 617145 treatment enriched WRN in the chromatin fraction of human cells (Aggarwal et al., 2013b). It remains to be seen if (or what) other DNA replication/repair factors are sequestered with WRN in the chromatin fraction due to the presence of the drug. This is particularly relevant as protein hijacking may contribute to the cytotoxicity of the WRN helicase inhibitor.

Further studies with NSC 617145 revealed that the drug is even more toxic in human cells that are doubly deficient in the FA pathway and DNA Protein Kinase C (PK_cs_), a DNA damage sensor and phosphorylating enzyme which is implicated in non-homologous end-joining (NHEJ) pathway of DSB repair (Aggarwal et al., 2013a). This finding suggests that the toxicity imposed by WRN helicase inhibition in the context of ICL-induced DNA damage does not derive from a self-imposed deleterious NHEJ pathway. Moreover, the results provide further evidence that WRN helicase inhibitors can induce synthetic lethality via a genetic-based and/or chemically induced mechanism. These results, along with those from other studies in the DNA damage response/DNA repair field, continue to spark interest in the development of anti-cancer strategies that exploit the genetic background of a tumor as well as drug combinations that together might overwhelm that resistance of tumors to mono-therapies. Moving toward a pre-clinical application is a priority in the DNA repair community, which will require advancement through model genetic systems and *in vivo* applications.

It seems likely that small molecule helicase inhibitors like those directed against WRN will operate in a manner that is dependent on the genetic background of the tumor. WRN, like other human RecQ helicases, is typically up-regulated in its expression in various cancer cell lines; moreover, their down-regulation by RNA interference has been shown to cause decreased proliferation (Brosh, 2013). Therefore, inhibition of WRN function may represent a useful strategy to compromise rapidly dividing cancer cells dependent on WRN to deal with replicative lesions. Analysis of the NCI 60 cancer cell database did not show a strong correlation between WRN protein level and sensitivity to NCS 19630 (Aggarwal et al., 2011), indicating a more complex scenario. Nonetheless, non-cancerous breast epithelial cells or normal fibroblasts were found to be resistant to NSC 19630 (Aggarwal et al., 2011), suggesting that a therapeutic threshold for the WRN inhibitor may come into play. Interestingly, it was reported that the susceptibility of breast cancer cells to killing by camptothecin (CPT) correlated with CPT-induced WRN degradation (Shamanna et al., 2016). Exposure to the WRN helicase inhibitor NSC 617145 was also observed to cause WRN degradation (Aggarwal et al., 2013b), suggesting that the anti-proliferative effects of compounds that target WRN or protein partners with which it interacts (e.g., topoisomerase I) is a causative factor. Further studies in this area may help to elucidate strategies to target tumors by exploiting their genetic background and negatively affecting the activity as well as the stability of DNA repair protein targets with small molecules.

### BLM

Bi-allelic mutations in the *BLM* gene result in Bloom's syndrome (BS) characterized by a pronounced predisposition to all types of cancer and certain features of accelerated aging (de Renty and Ellis, 2017). A prominent form of chromosomal instability used to clinically diagnose BS is elevated sister chromatid exchange (SCE) that is attributed to defects in recombinational repair and a poor replication stress response. The BS helicase (BLM) shares sequence homology within the conserved ATPase/helicase core domain of WRN and RecQ orthologs. In addition, BLM (as well as WRN) contains a conserved RecQ C-terminal (RQC) region that bears Zn^2+^-binding and winged helix (WH) domains and a Helicase RNase D-like C-terminal (HRDC) domain (see below). The RQC is implicated in structure-specific DNA binding and protein interactions of WRN and BLM (Estep and Brosh, 2017), and the HRDC is implicated in ligand-induced conformational changes in BLM (Newman et al., 2015).

To gain greater insight to BLM's molecular and cellular roles in DNA metabolism, a HTS of greater than 350,000 compounds from the Molecular Libraries Small Molecule Repository (MLSMR) was undertaken to identify inhibitors of BLM-catalyzed helicase activity on a fluorometric-labeled forked duplex DNA substrate (Nguyen et al., 2013). For this assay, a recombinant truncated version of BLM containing the helicase core domain but lacking 119 amino acids in the N-terminus and 635 residues in the C-terminus was used. Of the positive hits for BLM helicase inhibition, one compound was optimized for its medicinal chemistry properties (e.g., solubility, cell permeability), leading to ML216 (Table 1) as a lead candidate for further studies. The IC_50_ for ML216 inhibition of helicase activity catalyzed by the BLM helicase domain fragment or full-length BLM was determined to be in the low micromolar range. Interestingly, ML216 was much less effective in inhibiting branch-migration of synthetic Holliday Junction (HJ) or mobile D-loops DNA substrates, as well as a G-quadruplex DNA substrate, with an IC_50_ value of ~ 50 μM (Nguyen et al., 2013). The differential effect of ML216 on BLM catalytic activity with these different DNA substrates would suggest that the compound affects BLM in a unique manner as it unwinds forked duplex DNA, and that BLM operates by distinct DNA structure-specific mechanisms during DNA unwinding and branch-migration, as suggested by previous studies (Mazina et al., 2012). Although ML216 displayed specificity for helicase inhibition based on results from assays with UvrD, RECQL1, and RECQL5 helicases (IC_50_ > 50 μM), the small molecule inhibited forked duplex DNA unwinding by a WRN helicase domain fragment or full-length WRN at significantly lower drug concentrations (Nguyen et al., 2013). The IC_50_ value for inhibition of forked duplex DNA unwinding by full-length WRN was only 1.7-fold greater than IC_50_ value for inhibition of unwinding by full-length BLM on the same partial duplex DNA substrate. This raises the possibility that ML216 binds to both BLM and WRN through their conserved ATPase/helicase core, RQC, or HRDC domains; however, biophysical mapping studies are required to address this. ML216 was shown to inhibit BLM binding to single-stranded DNA or forked duplex DNA (Nguyen et al., 2013), suggesting that the compound inhibits BLM-catalyzed DNA unwinding by interfering with its DNA binding function, but an analysis of ML216's effect on WRN DNA binding was not reported in the study.

A structurally related analog of ML216 (5-(pyridin-4-yl)-1,3,4-thiadiazol-2-amine derivative, designated as compound 33) was achieved through medicinal chemistry efforts focused on structure-activity relationships (SAR) (Rosenthal et al., 2013). Like ML216 (Nguyen et al., 2013), compound 33 inhibited BLM helicase activity and single-stranded DNA binding, consistent with its non-ATP competitive inhibition of DNA-dependent BLM ATPase activity (Rosenthal et al., 2013). Compound 33 showed a greater selectivity for inhibition of BLM helicase activity over WRN compared to ML216. Although compound 33 also inhibited single-stranded DNA binding by WRN, its effect was not quite as potent as that observed for BLM DNA binding.

From a preclinical perspective, an initial litmus test for the medicinal development of a small molecule DNA repair inhibitor is its activity in cell-based models. ML216 was observed to inhibit proliferation of human SV40-transformed skin fibroblasts in a BLM-specific manner, i.e., the presence of BLM in the isogenic cell line was required for ML216 (12.5 or 50 μM) to inhibit proliferation in the 48 or 72-h time-period (Nguyen et al., 2013). Because elevated SCE is such a pronounced phenotype of BS, the effect of BLM small inhibitors ML216 (Nguyen et al., 2013) and compound 33 (Rosenthal et al., 2013) on this form of chromosomal instability in human cells was assessed. Both BLM inhibitors induced SCE in BLM-positive, but not BLM-negative cells, consistent with a BLM-dependent effect. Pre-exposure to 50 μM ML216 for 24-h sensitized human cells to the DNA polymerase inhibitor aphidicolin in a BLM-dependent manner (Nguyen et al., 2013). A 48-h pre-exposure to ML216 caused a greater frequency of γ-H2AX foci (a marker of DSBs) induced by MMC in a BLM-dependent manner as well. Thus, the results suggest that the BLM helicase inhibitor ML216 as well as compound 33, like the WRN helicase inhibitors NSC 19630 and NSC 617145, behave in a dominant-negative fashion, relying on their helicase target to cause anti-proliferative and DNA damage-inducing effects. However, both ML216 (Nguyen et al., 2013) and compound 33 (Rosenthal et al., 2013) impaired DNA binding by BLM [whereas, the WRN inhibitor NSC 19630 did not appreciably affect WRN DNA binding at drug concentrations in which significant helicase inhibition was observed (Aggarwal et al., 2013b)], suggesting that the BLM inhibitors may not trap BLM helicase protein on genomic DNA in cells. This is contrasted to NSC 617145, which was reported to enrich WRN's association with chromatin (Aggarwal et al., 2013b).

### DNA2

DNA2 is a protein with dual helicase and endo-/exo-nuclease activities originally discovered in yeast to play an important role in processing of DNA replication intermediates (Budd et al., 1995, 2000). These advancements laid the foundation for studies of DNA2 in human cells, which also revealed its importance in DNA metabolism. Emerging evidence indicates that in addition to DNA2's involvement in Okazaki fragment processing (Kang et al., 2010b), the helicase-nuclease is important for DNA end-processing as an early step in DSB repair (Symington, 2016) and nucleolytic processing of stalled or regressed forks that arise during replication stress (Thangavel et al., 2015). These findings, coupled with observations that DNA2 is overexpressed in various cancers, has made DNA2 an attractive candidate for inhibition as a strategy for cancer therapy (Jia et al., 2017). Indeed, several groups have reported that DNA2 depletion by RNA interference causes the reduced proliferation of cancer cells (Jia et al., 2017). Thus, DNA2 may be suitable for chemical inhibition by small molecules that inhibit its catalytic nuclease and/or helicase function.

A HTS with a fluorometric DNA substrate and yeast DNA2 was employed to search for inhibitors of the enzyme's nuclease activity (Kumar et al., 2017). From this screen of ~50,000 compounds, a couple of compounds (NSC 5195242, NSC 105808) were identified that could inhibit yeast and human DNA2 nuclease in a specific manner (by its negligible effect on other nucleases tested) (Table 1). However, these compounds were not tested on nuclease-dead versions of DNA2 to assess if they affected DNA2 helicase activity. It was found that NSC 105808 did not affect DNA2 ATPase (Kumar et al., 2017). NSC 105808 negatively affected proliferation of human bone osteosarcoma U2OS cells, and its anti-proliferative effect was suppressed by ectopic expression of DNA2 at a level 1.5–2.0-fold greater than endogenous DNA2, leaving the authors to propose that DNA2 is the target of the compound (Kumar et al., 2017). However, NSC 105808 was not tested on DNA2-deficient or DNA2-depleted cells, so a comparison to the mechanisms of action for the reported WRN- or BLM-specific helicase inhibitors which impaired cell proliferation in a manner that was dependent on the presence of either RecQ helicase in human cells has not been done. Nonetheless, it may be speculated that a small molecule which causes the trapping of a helicase on the DNA, such as WRN inhibitor NSC 617145 (Aggarwal et al., 2013b), may behave quite differently from a DNA2 nuclease inhibitor such as NSC 105808. Further studies are warranted to characterize the cell-based effects of chemical DNA2 nuclease inhibitors.

Both DNA2 nuclease inhibitors NSC 105808 and NSC 5195242 inhibited DNA end processing in a reconstituted system (Kumar et al., 2017). Moreover, NSC 105808 was observed to diminish DNA end-resection and HR in human cells. In addition, NSC 105808 suppressed the sensitivity of FANCD2 –/– cells to cisplatin, like the effect of DNA2 depletion (Kumar et al., 2017), suggesting that the compound targets DNA2. In several different cancer cell models, the DNA2 inhibitor suppressed proliferation of cancer cells with oncogene-induced replication stress (Kumar et al., 2017), suggesting a potential avenue of further exploration for cancer therapy. Studies with DNA2 inhibitors applied to genetic organisms and mouse xenografts that serve as good preclinical models will help to address the usefulness of these compounds for further development.

### Mcm2-7

The mini-chromosome maintenance protein 2-7 (Mcm2-7) is a well conserved hexameric DNA helicase that plays an essential role in DNA replication by unwinding the parental duplex strands to be copied (Abid Ali and Costa, 2016). The observations that certain mutations in the Mcm helicase subunits are associated with cancer and that Mcm is over-expressed in cancer cells supports the idea that this hexameric helicase complex is a suitable target for cancer therapy (Neves and Kwok, 2017). Currently, only very limited work has been done to identify inhibitors of DNA unwinding by Mcm complexes. It was determined that helicase activity catalyzed by the Mcm467 subcomplex was inhibited by heliquinomycin (Ishimi et al., 2009); furthermore, this compound decreased proliferation of cancer cells grown in culture (Toyokawa et al., 2011) (Table 1). More recent efforts in this area led to the identification of a fluoroquinoline antibiotic known as ciprofloxacin (previously shown to deter the catalytic function of topoisomerase II) as an inhibitor of the Mcm2-7 helicase (Simon et al., 2013) (Table 1). Ciprofloxacin was shown to inhibit the growth of yeast cultures, and one of the mcm mutant strains tested was resistant to the compound, suggesting that Mcm2-7 is a target of the drug.

## Successful virtual screens to discover helicase inhibitors

We expect these seminal studies to be followed by new compound inhibitors designed by molecular docking with recently solved DNA helicase structures. Most of the docking programs perform two basic operations, “docking” and “scoring.” Ligands are docked into the protein structure to predict most possible conformations of the protein-ligand complexes, particularly the conformations of the ligands bound to the binding pockets of the target protein. In the second operation, using a scoring function, the binding affinities of the individual ligands to the target protein in each conformational state are calculated and thus multiple ligands are ranked according to their respective docking score. In the following sections, we will discuss advances to identify helicase inhibitors by virtual screening.

### DDX3

Resistance of HIV-1 to the commonly used anti-HIV drugs is often due to drug-induced acquired mutations in the viral enzymes. Targeting host cell cofactors holds immense therapeutic potential because they are less susceptible to drug-induced mutability compared to the viral enzymes (Kwong et al., 2005). Cellular RNA helicases (e.g., RNA Helicase A (RHA), RNA Helicase 116 (RH116), DEAD-box helicases DDX1 and DDX3) play crucial roles in HIV-1 replication inside the host cells and may represent good targets, provided that cytotoxicity is not a factor. Cell-based screening of a series of ring-expanded nucleoside (REN) analogs identified a potent small molecule inhibitor (CID 44586781) of DDX3, an ATP-dependent RNA helicase required for exporting HIV-1 RNA from the nucleus to cytoplasm (Yedavalli et al., 2008). CID 44586781 was effective in suppressing HIV-1 replication in macrophages and T cells without imparting significant cytotoxicity *in vivo*. Notably, DDX3 was one of the first helicases subjected to the structure-based design of small molecular inhibitors using a molecular modeling approach (Maga et al., 2008). Using the crystal structure of DDX3 bound with AMP, a potential inhibitor of the enzyme's ATPase activity, designated RBD4 (Table 1), was identified by pharmacophoric modeling and subsequent molecular docking-based virtual screening of compound libraries. In cell-based assays, the small molecule inhibitor was found to be effective in inhibiting HIV-1 replication, thereby strengthening the power of the docking approach. Optimized inhibitors that interfere with DDX3 RNA binding and helicase activity were also identified by precise homology modeling followed by high-throughput molecular docking (Radi et al., 2012). Although there are other cellular helicases which could serve as potential anti-HIV1 drug targets, only DDX3 has been successfully targeted by small molecule inhibitors so far. Therefore, structure-based design and virtual screening approaches targeting additional helicases involved in HIV-1 replication may aid in the development of more potent and effective inhibitors.

### NS3

The Hepatitis C virus (HCV) NS3 helicase plays a key role in HCV replication and has been an attractive target for developing antiviral drugs (Frick, 2007). A potent inhibitor of NS3 helicase was successfully identified by exploiting its crystal structure using molecular docking-based virtual screening (Chen et al., 2009). The blue soluble HT dye that docked into the NS3 ATP binding site was found to inhibit its helicase activity as measured by a FRET-based assay (Chen et al., 2009). The co-crystal structure of the compound with NS3 was subjected to a small chemical fragment-based virtual screening search, leading to the discovery of a novel triphenylmethane derivative (Compound 12) that suppressed HCV replication in host cells (Chen et al., 2009). More recently, an *in silico* small molecule docking screen was used to identify an anti-helminthic drug (ivermectin) as a potent inhibitor of NS3 helicase activity; furthermore, ivermectin suppressed replication of common flaviviruses in cultured cells (Mastrangelo et al., 2012).

### DNA2

The crystal structure of murine DNA2 bound to a short (15 nt) single-stranded DNA molecule revealed a unique mechanism of nucleolytic processing of DNA strand in which single-stranded DNA threaded through a central tunnel where it is bound by both the nuclease and helicase domains (Zhou et al., 2015). Although this murine DNA2 crystal structure has not yet been exploited for molecular docking of small molecules, an alternative virtual screening approach was used (Liu et al., 2016). The researchers employed the crystal structure of yeast Upf1-RNA U15 complex and human Upf1-ADP complex (because they share high sequence identity with DNA2) to generate a stable homology model of human DNA2 bound to single-stranded DNA and then predicted potential druggable sites on the protein surface by docking a set of FDA-approved drug molecules. The most favorable docking pocket with the maximum score and DNA binding affinity was then subjected to HTS using a large NCI Developmental Therapeutics Program (DTP) library of small molecules. The approach led to the successful discovery of a lead compound (C5) that was predicted to bind to the DNA binding sites within the helicase domain.

The helicase domain-interacting molecule, designated C5 (Table 1), impaired nuclease, ATPase, and helicase activities of DNA2 (Liu et al., 2016). C5 inhibited proliferation of multiple cancer cell lines originating from breast, colon, prostate, or lung. Depletion of DNA2 in the breast cancer cell line MCF7 suppressed the anti-proliferative effect of C5, suggesting that DNA2 is the target of the compound *in vivo*. Consistent with this finding, embryonic stem cells from dna2^−/−^ mice were also resistant to C5. The authors did not assess if C5 caused sequestration of DNA2 on DNA, but this would be a worthwhile experiment to address the cytotoxicity of the DNA2-interacting compound.

In further cell-based assays, C5 was shown to inhibit single-stranded DNA annealing and HR, and this effect was likely due to a negative effect on DNA2-mediated end resection because RPA foci formation was reduced after CPT exposure in the C5-treated cells (Liu et al., 2016). To assess the effect of C5 on DNA2's involvement in fork stabilization/restart, DNA fiber assays were performed with cells exposed to the replication inhibitor HU or low levels of CPT. The results from these assays indicated that C5 prevents normal restart of stalled replication forks. Furthermore, C5 prevented over-resection of stalled forks, suggesting the compound prevents DNA2's catalytic activities from processing stalled or regressed forks. Finally, it was shown that C5 sensitized cancer cells lines to various chemotherapeutic agents including a PARP inhibitor and CPT.

## New DNA helicase structures provide future targets for molecular docking

Recent discoveries of DNA helicase crystal structures have been informative from a mechanistic perspective and suggest that the development of specific helicase inhibitors using rational drug design approaches will accelerate in the future. Given the importance of RecQ helicases in genomic stability and their proposed differences and overlap in function, efforts to dock compounds on functionally distinct and less conserved domains of RecQ helicases is warranted and may provide useful tools to not only explore RecQ biological functions but also develop chemotherapy drugs against the helicase targets. In the following sections, we will discuss some key structural features of RecQ helicases and a Fe-S helicase (XPD) which may be exploited for drug development.

### RECQL1

**Figure 3:**
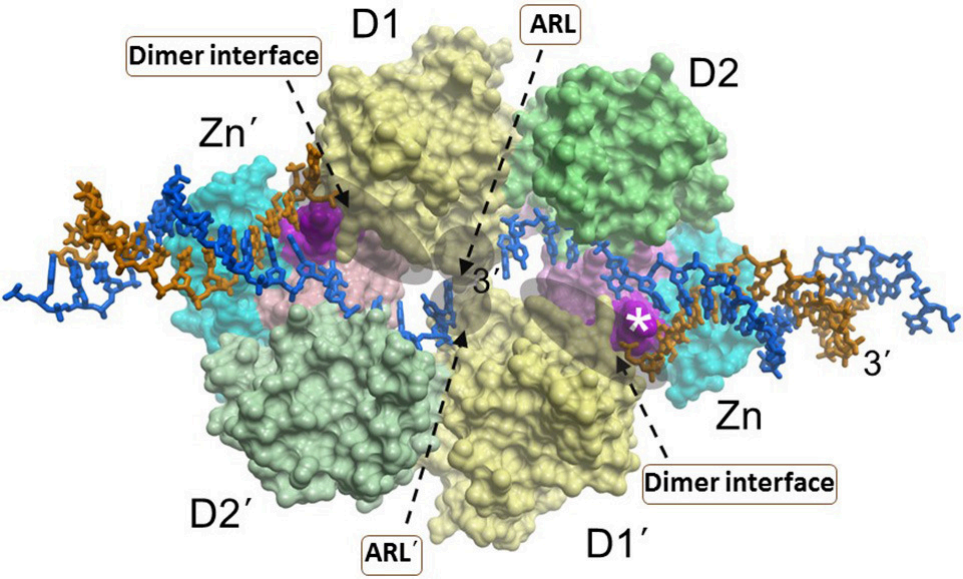
A dimer of RECQL1 molecules, bound to two forked DNA molecules. The first RECQ1 monomer is marked with standard lettering, the second monomer is with primes. The two RecA binding domains (D1, D2) and Zn^2+^ binding domain (Zn) are indicated. The white asterisk denotes the strand-separating beta hairpin. 3' end of DNA strand is indicated. The locations of the conserved aromatic-rich loops (ARL) implicated in the coupling of single-stranded DNA binding to ATP hydrolysis are indicated by the gray shadow. The region comprising the dimer interface between RECQL1 monomers is indicated by gray shadow. Docking small molecules at the ARL or dimer interface may provide a strategy to modulate RECQL1's catalytic function. Image was modified from one kindly provided by Dr. Opher Gileadi, University of Oxford.

Like the other RecQ helicases, RECQL1 possesses two conserved RecA motor domains positioned such that the nucleotide binds within the cleft (Pike et al., 2009, 2015; Lucic et al., 2011) (Figure 3). High-resolution X-ray crystal structures of human RECQL1 bound to DNA and biochemical studies by the Gileadi and Vindigni labs revealed that the conserved WH domain (adjacent to the Zn^2+^ binding domain) bears a prominent β-hairpin structure with a tyrosine residue (Y564) at the tip which acts as a unique strand separating pin; this β-hairpin is also found in WRN and BLM helicases, but theirs are considerably shorter than the one located in RECQL1 (Pike et al., 2015). The intimate interaction of the RECQL1 strand-separating β-hairpin with the DNA branchpoint of the single-stranded DNA-double-stranded DNA junction suggests a relevant target for molecular docking by small molecules. Nonetheless, conservation of the β-hairpin among other DNA helicases may compromise its utility as a drug target due to specificity issues. However, RECQL1's strand-separating pin is buttressed by a protein dimer interface required for optimal duplex DNA unwinding; furthermore, RECQL1 also forms a tetramer that is implicated in HJ branch-migration. We conjecture that small molecules which dock at sites of critical contact points for oligomerization (e.g., dimer interface) might effectively modulate RECQL1's assembly state that would have dramatic consequences for biochemical and cellular function (Figure 3). In addition, RECQL1 has a conserved aromatic-rich loop (ARL) within the ATPase/helicase core domain that couples single-stranded DNA binding to ATP hydrolysis; the critical nature of RECQL1's ARL for its helicase activity, as revealed by site-directed mutagenesis studies (Banerjee et al., 2015), suggests another structural target to pharmacologically modulate its catalytic activity (Figure 3). However, the fact that all five human RecQ helicases possess the conserved ARL (Estep and Brosh, 2017) raises doubt if interaction specificity of a small molecule would be easily achievable unless mitigating factors are addressed with sophisticated molecular docking approaches (see below).

### BLM

**Figure 4:**
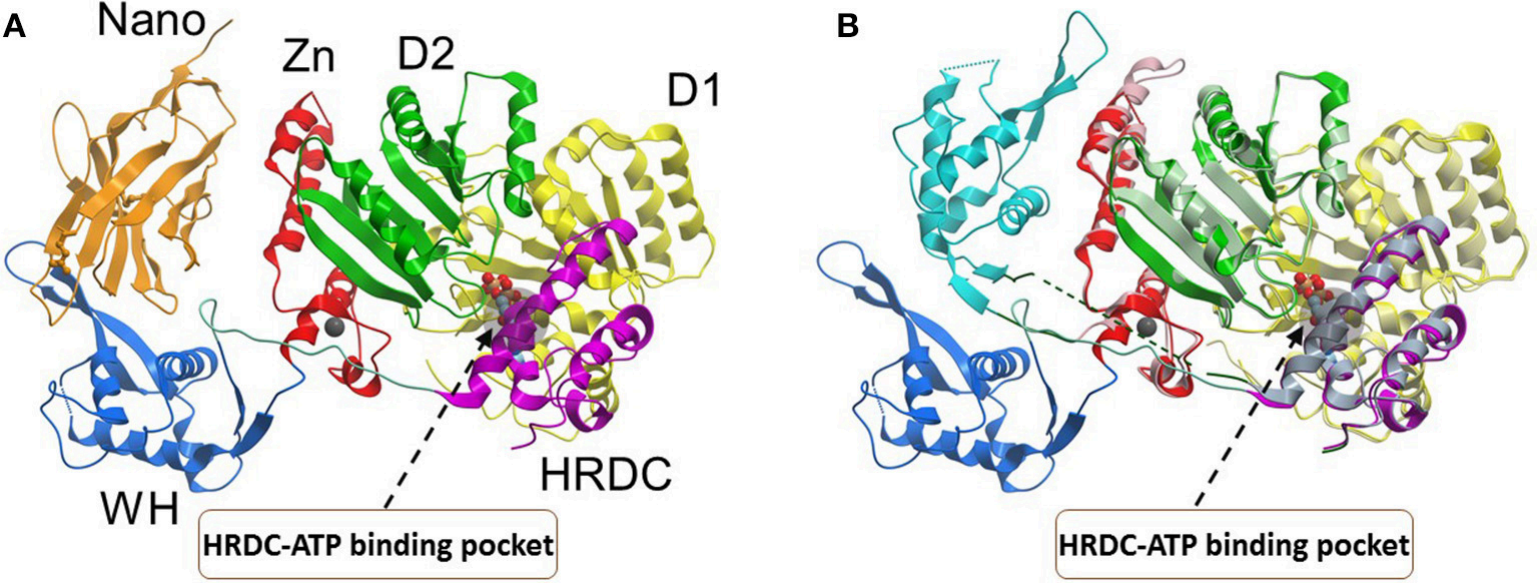
Conformations of the BLM helicase core and winged helix domains**. (A)** A co-crystal structure with a nanobody (orange), which shifts the WH domain out of its helicase-active position (PDB:4CDG). **(B)** A superposition of the nanobody-bound conformation (WH domain in blue, nanobody omitted), with a DNA-bound conformation (WH domain in cyan, DNA omitted; PDB:4CGZ). The RecA domains (D1, D2) and the conserved HRDC are indicated with a region of the HRDC residing close to the ATP binding pocket shown by gray shadow. Small molecules which bind to the HRDC may modulate ATP binding and/or hydrolysis by BLM. Image was modified from one kindly provided by Dr. Opher Gileadi, University of Oxford.

A recent BLM-DNA crystal structure solved by the Gileadi lab provided fresh insight to its DNA unwinding mechanism, suggesting a base-flipping action that is critical for duplex strand separation (Newman et al., 2015) (Figure 4). The mobility of the WH domain evident from the BLM structures suggests that a small molecule which docks in an allosteric site controlling the relative orientation may alter BLM's DNA unwinding mechanism. In addition, a new significance to the auxiliary HRDC domain found only in the human BLM and WRN helicases was ascribed. The HRDC domain was previously implicated in specialized DNA substrate recognition/binding and protein interaction for BLM and WRN [for review, see (Estep and Brosh, 2017)]. Moreover, the BLM HRDC domain is functionally important in double HJ dissolution, a reaction catalyzed by a BLM-topoisomerase complex that is believed to help suppress SCE, a characteristic feature of Bloom's syndrome (Wu et al., 2005). The new BLM structural data indicated a close residence of the HRDC domain to the nucleotide-binding pocket formed by the cleft between the two RecA domains (Newman et al., 2015), suggesting a structural arrangement that might be affected by HRDC-interacting small molecules (Figure 4). Compounds that interfere with the interaction of the HRDC with the RecA cleft would be predicted to disrupt the overall catalytic ATPase cycle of BLM, which in turn would affect its helicase activity. Thus, the BLM-DNA structure provides a framework for rational design of BLM-specific inhibitors that should deeply perturb its mechanism of action in cells.

### RECQL4

**Figure 5:**
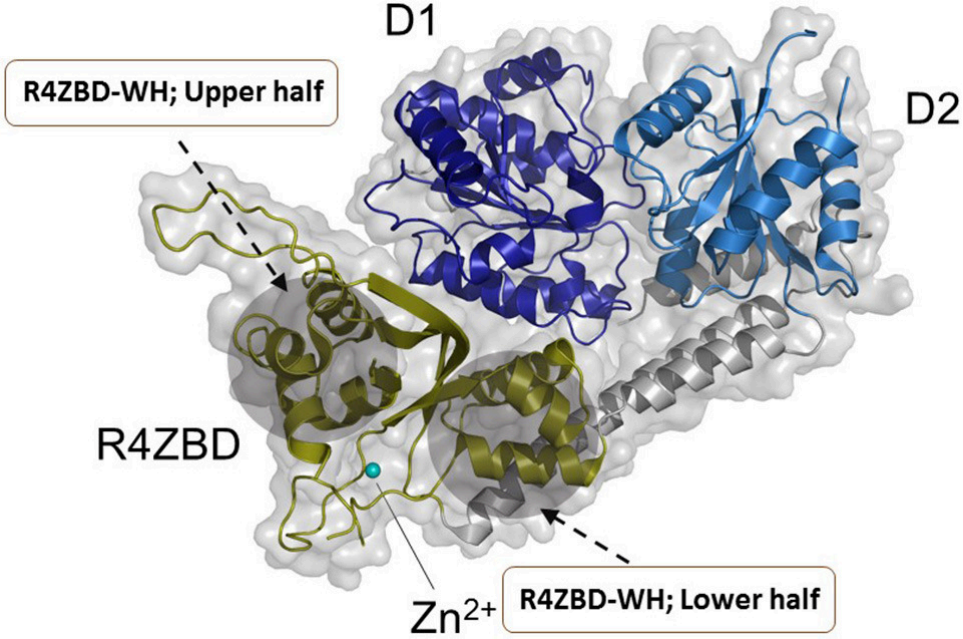
Structure of human RECQL4 (residues 449-1111). The ATPase domain, comprising HD1 and HD2, are shown in dark blue and light blue, respectively. RECQL4 features a structurally unique domain, termed RECQL4-Zn^2+^-binding domain (R4ZBD), shown in olive. The R4ZBD coordinates a Zn^2+^-ion (cyan sphere). The gray shadow regions represent the upper and lower halves of the R4ZBD that may be suitable for molecular docking of small molecules to the unique Zn^2+^ binding domain of RECQL4. RECQL4 harbors the Sld2-homology domain at its N-terminus (not shown). Image was modified from one kindly provided by Drs. Sebastian Kaiser and Caroline Kisker, University of Wuerzburg.

Hereditary mutations in *RECQL4* result in three genetically distinct diseases known as Rothmund-Thomson syndrome, Baller-Gerold syndrome, and RAPADILINO syndrome (Lu et al., 2017). While the *RECQL4* gene product is a DNA helicase, the unwinding activity catalyzed by the purified recombinant RECQL4 helicase protein measured *in vitro* on conventional duplex DNA substrates is relatively weak (Macris et al., 2006; Xu and Liu, 2009). The limited unwinding activity of RECQL4 may be due to its strong annealing activity and its protein architecture as it lacks the classical Zn^2+^ binding domain and WH domain found in most other RecQ helicases (Figure 5). However, as revealed by structural and biochemical studies from the Kisker lab, the C-terminus of RECQL4 contains a unique Zn^2+^ binding domain (R4ZBD) and a region sharing homology to two winged helices that are distinct from the RQC WH in other RecQs (Kaiser et al., 2017). The unique identity of RECQL4's C-terminal region, which was found to be important for DNA unwinding (Kaiser et al., 2017), suggests a potential site for molecular docking of small molecules in the upper or lower half of the R4ZBD-WH to modulate its catalytic function (Figure 5).

### RECQL5

**Figure 6:**
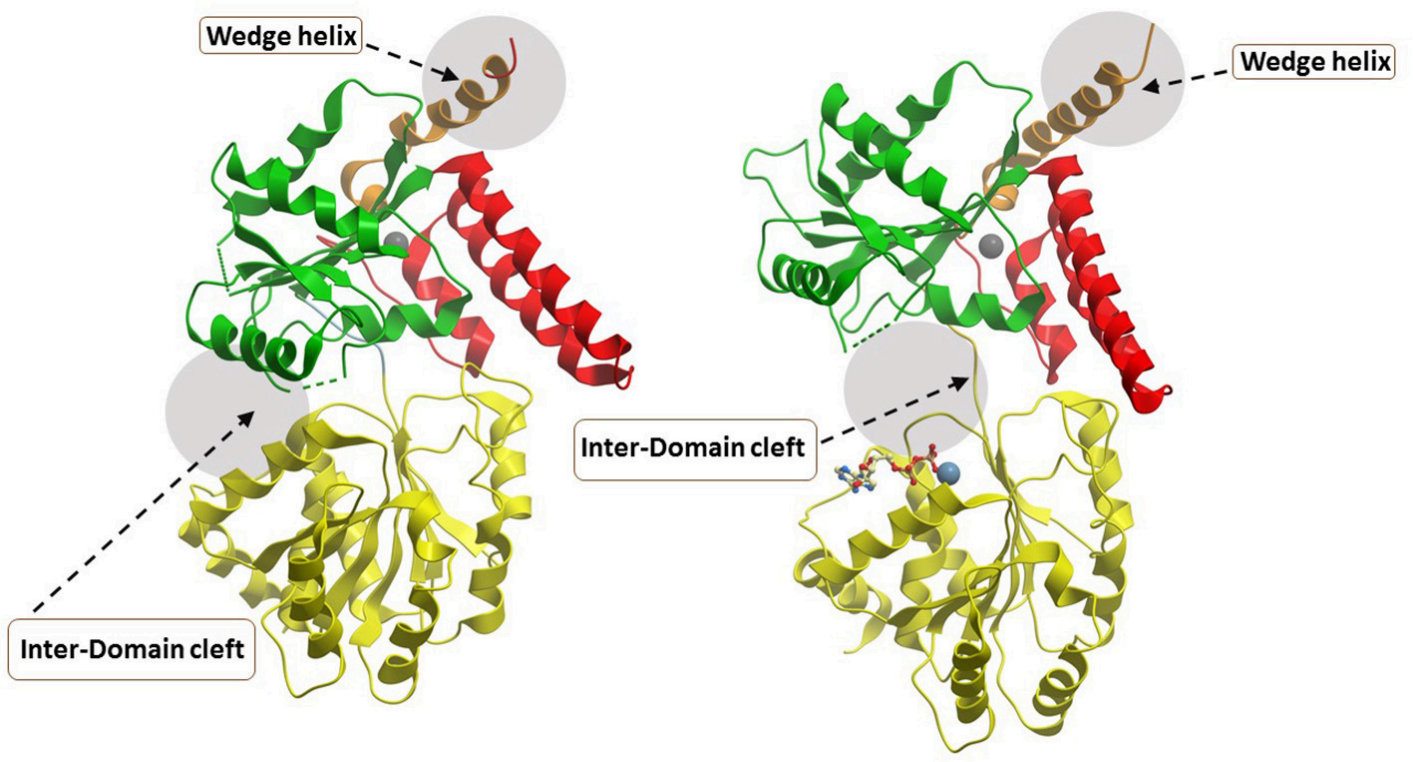
Closed and open conformations of RECQL5 helicase domain**. (A)** Closed state, no nucleotide (PDB ID: 5LB8); **(B)** Open state, with Mg^2+^-ADP (PDB: 5LB3). Note the absence of the WH and HRDC domains in the C-terminal region of the protein which can be found in WRN, BLM, and RECQL1. The unique “wedge” α-helix of RECQL5 critical for helicase activity is indicated and may be targeted by small molecules to modulate ATP-dependent strand separation. Also indicated by gray zone is the inter-domain cleft which widens significantly between the open and closed conformations. The inter-domain cleft may be a useful site for molecular docking of small molecule interfacial compounds that perturb open-closed conformational switches of RECQL5. Image was modified from one kindly provided by Dr. Opher Gileadi, University of Oxford.

The most recently solved structure of a human RecQ helicase was that of RECQL5 (Newman et al., 2017) (Figure 6). This work from the Gileadi lab showed that RECQL5 binds Zn^2+^ via the conserved domain found in RECQL1, WRN, and BLM and that RECQL5 possesses a unique adjacent α-helix with positively charged residues on its surface not found in the other human RecQ helicases. The unique RECQL5 α-helix is proposed to operate as a wedge analogous to the β-hairpin in RECQL1, WRN, and BLM, suggesting a potential RECQL5-specific domain to target with small molecules (Figure 6). From a molecular docking perspective, it is quite interesting that RECQL5 was demonstrated to exist in two distinct conformations (open and closed) that are regulated by nucleotide binding. Further studies may identify interfacial small molecule inhibitors that bind within the inter-domain cleft and lock it into the open or closed conformation (Figure 6). Screening for small molecules that affect the nucleotide-induced conformational switch of RECQL5 may be informative for further understanding mechanism. The aforementioned α-helix was implicated in DNA binding and site-specific mutagenesis revealed that it plays an important role in helicase activity. RECQL5-interacting compounds that affect the conformational freedom of the α-helix and other key structural elements would be anticipated to impact its catalytic functions and potentially modulate its functions in cells.

### XPD

Eukaryotic XPD is a component of the general transcription factor (TF)IIH complex that is implicated in both cellular transcription and nucleotide excision repair (NER) of damaged DNA (Kraemer et al., 2007). Mutations in the *XPD* gene are linked to genetic diseases characterized by premature aging and/or cancer predisposition including Trichothiodystrophy (TTD), Xeroderma pigmentosum, and Cockayne's syndrome (Lehmann, 2001). High-resolution crystal structures of XPD helicase and the data obtained from the associated biochemical and mutational studies over the past decade provided significant mechanistic insights about the function of this important class of Fe-S helicases (Liu et al., 2008; Wolski et al., 2008; Kuper et al., 2012; Abdulrahman et al., 2013). Crystal structures of archaeal homologs of XPD revealed that in addition to two canonical RecA motor domains, the structure contains a Fe-S cluster and Arch domain. In its proper conformational state, the XPD Fe-S cluster remains tightly connected to the ATP binding/hydrolysis domain. It has been proposed that the wedge-like structure formed by the Fe-S and Arch domains facilitates unwinding of duplex DNA during ATP-driven translocation of the enzyme. Furthermore, in addition to its essential role in helicase activity, mutational studies confirmed that Fe-S cluster is structurally important to maintain proper folding and stability of the XPD protein (Rudolf et al., 2006; Fan et al., 2008; Pugh et al., 2008).

**Figure 7:**
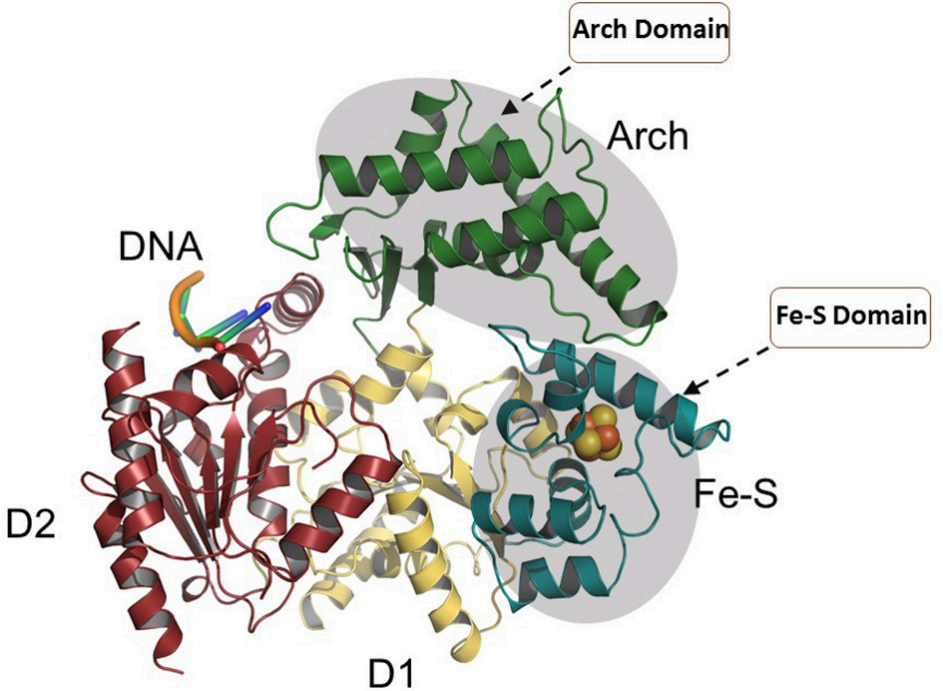
Overall structure of the taXPD-DNA complex. Shown are the two RecA-like domains in yellow orange and ruby, the FeS cluster domain in deep teal, and the Arch domain in forest green. The DNA is shown in orange/yellow/blue. The Arch and Fe-S domains, both implicated in strand separation and only found in Fe-S helicases, are proposed sites for docking small molecules to modulate helicase function. Image was modified from one kindly provided by Drs. Jochen Kuper and Caroline Kisker, University of Wuerzburg.

More functional insights came from the crystal structure of XPD from *Thermoplasma acidophilium* (taXPD) in complex with DNA solved by the Kisker lab (Kuper et al., 2012) (Figure 7). The taXPD-DNA complex structure, combined with biochemical and mutational analyses from their lab (Kuper et al., 2012) and Spies' (Pugh et al., 2012), has begun to elucidate the underlying mechanism for XPD's DNA translocation polarity, thereby providing insight into the role of the helicase during NER. Apart from the Fe-S cluster domain, the Arch domain of XPD also has been shown to be critical for its DNA binding and strand separating activities. Introduction of a TTD-linked point mutation (XPD-C259Y) or deletion of the entire Arch domain (XPD-ΔARCH) was found to impair DNA binding and helicase activity of XPD (Abdulrahman et al., 2013). Collectively, these studies suggest that Fe-S cluster and Arch domain play key roles in the unwinding mechanism and governing XPD functions during DNA damage repair. Targeting the Arch and Fe-S domains of XPD with small molecules may be valuable (Figure 7); however, these domains are conserved in other Fe-S helicases (FANCJ, RTEL1, DDX11), raising doubt if they would be specific. Nonetheless, given that DNA damaging chemotherapy drugs often introduce bulky lesions recognized by NER, it is reasonable to postulate that small molecule targeted inhibition of DNA unwinding by the XPD helicase (thought to be an early sensor or verifier of the DNA damage) would be useful to sensitize tumors to certain compounds used in anti-cancer treatments.

Further research in this area to solve structures of XPD with key DNA structural intermediates (e.g., DNA bubbles with site-specific damage), as well as structures of other Fe-S helicases, is likely to advance efforts in molecular docking and HTS for small molecules that modulate their functions. With the discovery of specific and potent inhibitors of Fe-S helicases, the co-crystal structures of helicase-DNA-small molecule complexes may elucidate key helicase interactions in DNA metabolic pathways. Molecular docking-based virtual screening should also be considered as a highly effective complementary approach toward the discovery of small molecule inhibitors of disease-linked DNA repair helicases.

## Challenges to development and application of helicase inhibitors

Although small molecule inhibitors of a few viral helicases showed preclinical and clinical success [e.g., the herpes simplex virus helicase-primase inhibitor Amenamevir (ASP2151) (Chono et al., 2010) and other drug candidates (Kleymann et al., 2002)], development of highly specific and pharmacologically effective helicase inhibitors is still challenging. In general, high throughput biochemical screening assays return very few hits and most often they fail to deliver expected biological effects in subsequent cell-based assays or display poor pharmacological outcomes [e.g., HPV helicase inhibitor (Faucher et al., 2004)]. As is the case for a significant number of small molecule inhibitors, bioavailability is likely to be one of the major obstacles to developing clinically effective DNA repair helicase inhibitors and target validation is required (Bunnage et al., 2013). Therefore, cellular bioavailability parameters including inhibitor aqueous solubility, nonspecific binding to the cell membrane and extracellular matrix, cellular uptake, intracellular metabolic stability, and biotransformation to an inactive secondary metabolite should be taken into consideration for the successful therapeutic exploitation of a newly identified promising drug (Frye, 2010; Workman and Collins, 2010), such as a lead molecule against a helicase protein. For example, although BLM inhibitors ML216 and its analog 33 were found to exhibit good general pharmacokinetic properties including clogP (computational method for measurement of drug hydrophilicity and lipophilicity properties), microsomal stability, and plasma stability, both compounds displayed low solubility and permeability suggesting that further optimization of the lead compounds is required (Rosenthal et al., 2013). In this scenario, sensitive assays such as a HPLC-MS based method to determine bioavailability of a given compound inside cells may prove fruitful to assess cellular uptake of helicase inhibitors (Teuscher et al., 2017).

Given the precise functions of helicases in nucleic acid metabolism, another consideration is if the helicase-interacting compound reaches its desired subcellular localization (i.e., nucleus, cytosol, mitochondrion) to bind its desired target helicase inside the respective cellular compartment. In order to get the most desirable therapeutic effect and to minimize the negative side effects, it is also very important to ensure that the helicase inhibitors are delivered specifically to their sites of action within the cells. Nuclear targetted delivery of these small molecule inhibitors might be achieved using nanoparticles coated with nuclear localization signal (NLS) (Kang et al., 2010a). Similarly, clinically approved nanoparticles should be considered to deliver the helicase inhibitors selectively to the target tumor sites at sufficient concentration to attain therapeutic efficacy.

One of the prime challenges associated with the development of helicase inhibitors is their relative potency and specificity. The inhibitors should be potent enough to exert their biological effects at minimal concentration. For example, Mcm2-7 helicase inhibitor ciprofloxacin (Simon et al., 2013) and BLM helicase inhibitor ML216 (Nguyen et al., 2013) were found to exhibit their bioactivity at relatively high concentrations (160–350 μM and 50 μM, respectively; see Table 1). Therefore, structural optimization of these hit compounds is warranted to obtain more potent leads. Moreover, the effect of ML216 is not entirely specific to BLM helicase because the compound also inhibits DNA unwinding by the sequence-related WRN helicase with a similar potency (Nguyen et al., 2013). Hence, promising hits identified for a target helicase from the initial screening should be further assessed for their potency as a function of drug concentration as well as for their specificity by determining their effects on other helicases. Substantial counterscreening of the newly identified potential helicase inhibitors is required before they can be reliably pursued in preclinical models.

A number of drug resistance mechanism are known to operate which contribute to tumor resistance (Holohan et al., 2013). These include: (i) drug efflux or alteration by activation or inactivation; (ii) alterations in the drug target by mutation or change in gene expression; (iii) repair of chemotherapy- or radiation-induced DNA damage; (iv) up-regulation or activation of compensatory signaling pathways; (v) cell death evasion (e.g., attenuated apoptosis). From the perspective of DNA helicase inhibitors, one of the basic principles is to compromise a helicase-dependent pathway of repair [(iii) above] to confer synthetic lethality; however, it is possible that another repair pathway or signaling pathway is elicited that compromises the efficacy of the helicase inhibitor. Certainly, it is plausible that even the functional redundancy between members of the RecQ or Fe-S helicases, for example, may contribute to helicase inhibitor resistance by a compensatory overlapping pathway. Other avenues of drug resistance, such as those mentioned above, may allow resistance to the anti-cancer effects of a helicase-directed drug inhibitor; however, little is known in this area because it is such a new field. The fact that tumors are often heterogeneous may allow cancer cell subpopulations survive under pressure from a cancer drug (Zahreddine and Borden, 2013), including one against a specific DNA helicase. These topics all deserve prioritized attention.

In terms of molecular docking approaches for the discovery of compounds that inhibit helicases and other DNA repair proteins, a significant challenge is the protein flexibility of the target in which intrinsic conformational states may compromise a good fit for the docking ligand (Tripathi and Bankaitis, 2017). Other mitigating factors including protein pocket architecture to accommodate the three-demonsional geometry of the ligand, ligand access (surface or protein interior), the role of structured water molecules in the ligand-target interaction, protonation, and ionization states of the protein: ligand system, and entropy considerations. Advances in artificial intelligence and machine learning algorithms provide new and innovative direction for structure-based drug design. These efforts, combined with high-resolution structures of helicase proteins, provide excitement for anticipated progress.

## Summary and future directions

**Figure 8:**
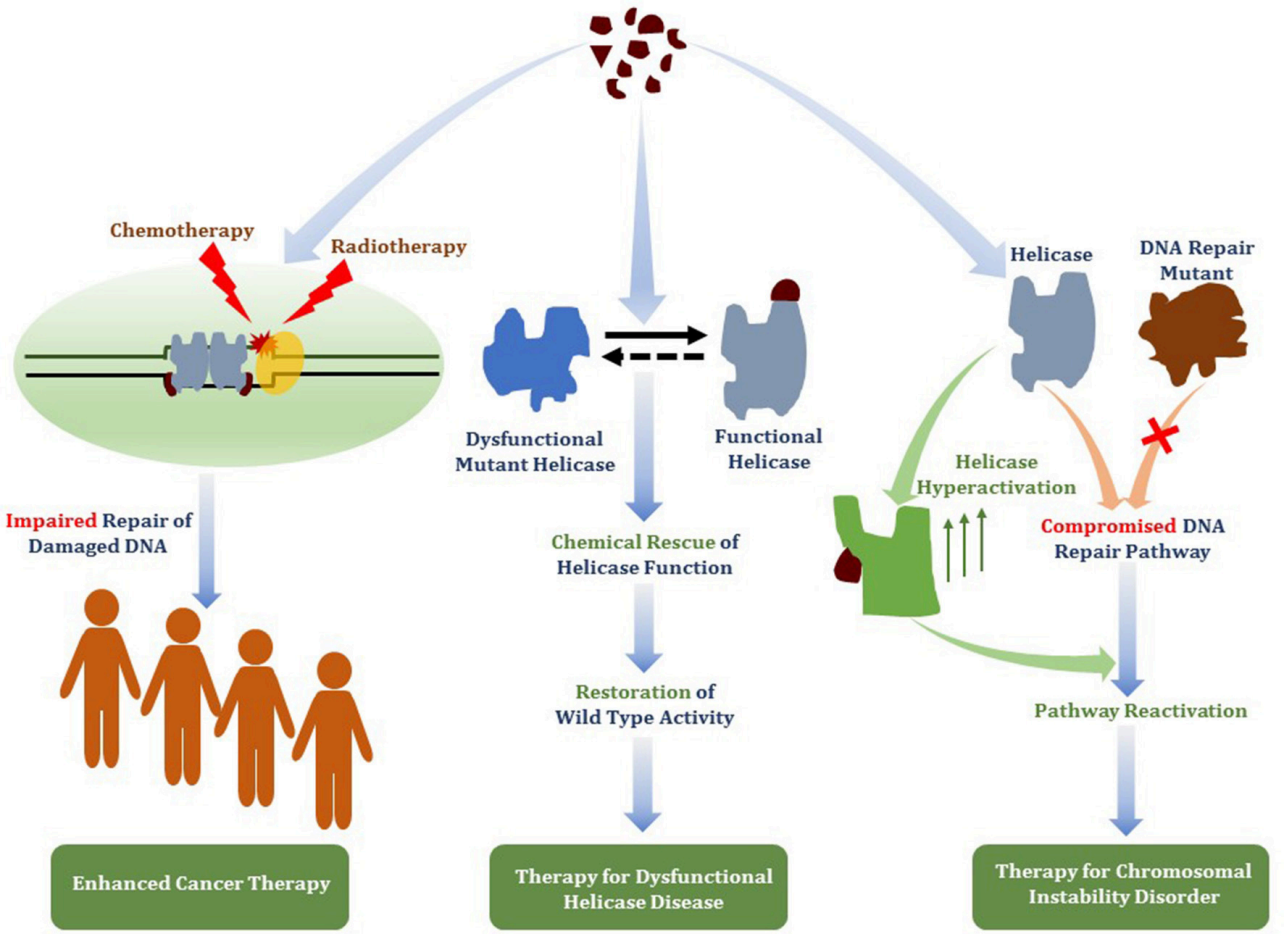
Therapeutic avenues for helicase-interacting small molecules. Compounds that bind DNA helicase proteins may be useful for (i) targeted helicase inhibition to deter DNA damage response pathways to enhance chemotherapy drug or radiation strategies (ii) chemical rescue of catalytically defective and/or misfolded helicase proteins due to missense mutations linked to hereditary chromosomal instability disorders, or (iii) functional activation of a wild-type helicase that has an overlapping role with a DNA repair pathway that is defective due to a genetic inactivating mutation linked to a DNA repair disorder.

DNA helicases are often recruited to sites of DNA damage or stalled replication forks. The very nature of their catalytic function to separate complementary DNA strands is imperative to a wide variety of DNA transactions that play instrumental roles in cellular DNA replication, recombination, repair, and transcription. Therefore, chemical modulation of the molecular functions of DNA helicases provides an approach to alter not only the efficiency or fidelity of transactions in nucleic acid metabolism but also affect cellular homeostasis, including the division rate of cancerous cells. As detailed in this review, DNA helicases join a larger class of DNA metabolic proteins that are considered as potential targets to augment radiation and chemotherapy strategies to combat cancer. Small molecule-induced trapping of DNA helicases may represent a generalized mechanism exemplified by certain topoisomerase and PARP inhibitors that exert poisonous consequences, especially in rapidly dividing cancer cells. An area that remains underexplored is the synergism between compounds that modulate different DNA proteins. This is particularly interesting from the perspective of DNA helicase inhibitors that might be combined with compounds that deter the functions of other DNA repair enzymes to enact targeted anti-proliferative and lethal effects in various cancer types (Figure 8). Just as chemical and genetic synthetic lethality has become more widely appreciated and better understood, we expect that pharmacological modulation of helicase function will move to the forefront as molecular motor DNA unwinding enzymes play such pivotal roles in nucleic acid metabolism and cross-talk with many cellular pathways. With the increased knowledge of structure-activity relationships from the solution of helicase structures, we anticipate that molecular modeling will provide a more readily accessible and informed pathway for the discovery of novel helicase-interacting compounds. An important challenge in the field will be the utilization of helicase-modulating drugs in preclinical models that will accelerate their implementation in therapeutic approaches.

A unique aspect of this review is to detail potential strategies to target helicase with small molecules using a structure-design molecular docking approach (Figure 2). We believe that virtual screening of small molecule libraries to identify compounds predicted to modulate helicase function will become main-streamed as more helicase structures (and their conformational states induced by ligand binding) become solved and computational strategies advance. An illustrative example of this approach was recently provided by the Berger lab. Lawson et al. observed that the structural interactions of nucleic acid or the antibiotic bicyclomycin with the same binding site in the hexameric RNA translocase/helicase Rho are distinguished from each other by the closed-ring (translocase competent) vs. open-ring (RNA binding defective) conformations of Rho, respectively (Lawson et al., 2016). This work is significant because it showed that nucleic acid substrate loading by a helicase could be modulated by a small molecule via a conformational switch in the enzyme that altered its ring-closure dynamics. Furthermore, the Rho-bicyclomycin study leads to the anticipation that other helicase-interacting small molecules may be identified virtually from compound libraries using a molecular docking approach that would be highly selective and mechanistically driven.

Although molecular compound inhibitors of DNA helicases or more generally DNA repair enzymes are increasingly discussed, conversations and research directions could also be directed toward small molecule chemical rescue of catalytically dysfunctional or misfolded helicase proteins as well as activation or up-regulation of wild-type helicase-catalyzed strand separation (Figure 8). Given the number of helicase missense mutations linked to hereditary disorders or associated with cancer (Suhasini and Brosh, 2013), and following the lead of other clinically relevant targets [e.g., chemical rescue of p53 missense mutations (Bullock and Fersht, 2001)], the prospect of finding treatments or cures for certain chromosomal instability disorders arising from catalytic deficiencies in helicase proteins is plausible. In another realm, helicase activation may have therapeutic value. For example, the possible functional overlap of DNA helicases [e.g., RecQ family members (Brosh, 2013; Croteau et al., 2014)] suggests that increased activity of one helicase may help to overcome the deficiency of another helicase or DNA repair protein (Figure 8). With molecular docking and high-throughput screens becoming more commonplace, the hypothesis that up-regulation of catalytic function by a helicase or DNA repair enzyme can rescue a helicase-deficient disease state may become testable in cell-based and pre-clinical models.

## Author contributions

AD and RB both contributed to writing original component and editing of the manuscript.

### Conflict of interest statement

The authors declare that the research was conducted in the absence of any commercial or financial relationships that could be construed as a potential conflict of interest.
